# Magnesium profiles in renal replacement therapy (rrt) on icu: citrate CVVH (CICVVH) vs. intermittent haemodialysis (IHD)

**DOI:** 10.1186/2197-425X-3-S1-A630

**Published:** 2015-10-01

**Authors:** N Billiet, L Merckx, M van der Laenen, P Vanelderen, W Boer

**Affiliations:** Intensive Care, Ziekenhuis Oost Limburg, Genk, Belgium

## Introduction

CiCVVH has become standard treatment for on ICU with AKI. Though regional citrate anticoagulation for CRRT is recommended as first line therapy in the AKI guideline by the KDIGO Group, IHD is practised in many ICU patients with AKI. Evidence for superiority for one technique over the other remains equivocal, though there is increasing evidence for better renal function preservation in CVVH. However, there is also evidence for increased non-recovering of renal function in ICU patients with AKI who have hypomagnesemia.

## Objectives

Goal is to compare magnesium profiles of ICU patients requiring RRT, treated with either ciCVVH or IHD.

## Methods

Over a 12 month period ICU patients were retrospectively studied for cases of AKI requiring RRT. Treatment episodes with ciCVVH (n=38) were compared to those treated with IHD (n=18 ). A number of patients underwent both ciCVVH and IHD. All magnesium values were analyzed from 12 hrs after initiation of RRT until cessation. Feeding in ICU patients is protocol-based and, though tailored to the diagnosis of AKI, does not differ based on mode of RRT. Magnesium supplementation generally takes place where values drop below 0.7 mmol/L. The substitution fluid used in ciCVVH contains 0.75 mmol/L. IHD fluids are magnesium free.

## Results

437 Magnesium values for patients on ciCVVH were analyzed (62.2%), 256 for IHD (36.4%). Magnesium values were significantly different in the 2 groups (mean (ciCVVH)=0,70 mmol/L, SD=0,22; mean (IHD) = 0.99 mmol/L, SD=0,20; p < 0,001). In ciCVVH, 265 of 437(61%) were below the normal threshold (normal values 0.7-0.9 mmol/L), in the IHD only 9 out of 256 (4%). Six values in the ciCVVH group were below 0.4 mmol/L, the threshold for severe hypomagnesemia, none in the IHD group.

## Conclusions

Magnesium values for patients on ciCVVH are significantly lower than IHD despite use of a Magnesium containing substitiion fluid. Dangerous values of hypomagnesemia can occur in ciCVVH. Review of magnesium testing and supplementation regimens in the ciCVVH group is warranted.

## References

[1] Alves SC, Tomasi CD, Constantino L, Giombelli V, Candal R, Bristot Mde L, Topanotti MF, Burdmann EA, Dal-Pizzol F, Fraga CM, Ritter C (2013). Hypomagnesemia as a risk factor for the non-recovery of the renal function in critically ill patients with acute kidney injury. Nephrol Dial Transplant.

